# Prognostic model of AU-rich genes predicting the prognosis of lung adenocarcinoma

**DOI:** 10.7717/peerj.12275

**Published:** 2021-10-08

**Authors:** Yong Liu, Zhaofei Pang, Xiaogang Zhao, Yukai Zeng, Hongchang Shen, Jiajun Du

**Affiliations:** 1 Institute of Oncology, Shandong Provincial Hospital, Cheeloo College of Medicine, Shandong University, Jinan, Shandong Province, China; 2 Institute of Oncology, Shandong Provincial Hospital Affiliated to Shandong First Medical University, Jinan, Shandong Province, China; 3 Department of Oncology, Shandong Provincial Hospital Affiliated to Shandong First Medical University, Jinan, Shandong Province, China; 4 Department of Thoracic Surgery, The Second Hospital of Shandong University, Jinan, Shandong Province, China; 5 Department of Thoracic Surgery, Shandong Provincial Hospital, Cheeloo College of Medicine, Shandong University, Jinan, Shandong Province, China

**Keywords:** LUAD, Prognostic signature, AU-rich genes, Immune, TCGA, GEO

## Abstract

**Background:**

AU-rich elements (ARE) are vital cis-acting short sequences in the 3’UTR affecting mRNA stability and translation. The deregulation of ARE-mediated pathways can contribute to tumorigenesis and development. Consequently, ARE-genes are promising to predict prognosis of lung adenocarcinoma (LUAD) patients.

**Methods:**

Differentially expressed ARE-genes between LUAD and adjacent tissues in TCGA were investigated by Wilcoxon test. LASSO and Cox regression analyses were performed to identify a prognostic genetic signature. The genetic signature was combined with clinicopathological features to establish a prognostic model. LUAD patients were divided into high- and low-risk groups by the model. Kaplan–Meier curve, Harrell’s concordance index (C-index), calibration curves and decision curve analyses (DCA) were used to assess the model. Function enrichment analysis, immunity and tumor mutation analyses were performed to further explore the underlying molecular mechanisms. GEO data were used for external validation.

**Results:**

Twelve prognostic genes were identified. The gene riskScore, age and stage were independent prognostic factors. The high-risk group had worse overall survival and was less sensitive to chemotherapy and radiotherapy (*P* < 0.01). C-index and calibration curves showed good performance on survival prediction in both TCGA (1, 3, 5-year ROC: 0.788, 0.776, 0.766) and the GSE13213 validation cohort (1, 3, 5-year ROC: 0.781, 0.811, 0.734). DCA showed the model had notable clinical net benefit. Furthermore, the high-risk group were enriched in cell cycle, DNA damage response, multiple oncological pathways and associated with higher PD-L1 expression, M1 macrophage infiltration. There was no significant difference in tumor mutation burden (TMB) between high- and low-risk groups.

**Conclusion:**

ARE-genes can reliably predict prognosis of LUAD and may become new therapeutic targets for LUAD.

## Introduction

Lung cancer is one of the most life-threatening diseases and possesses the considerable high morbidity and mortality (Siegel, Miller & Jemal , 2020). Lung adenocarcinoma (LUAD) is the most common histological subtype of lung cancer at present, accounting for 40% of all lung cancer cases (Hutchinson et al., 2019; Huang, Qu & Du, 2019). The current therapies include surgical resection, radiotherapy, chemotherapy, immune and targeted therapy. However, due to the rapid progression, early metastasis and lack of accurate biomarkers, most patients have an unfavorable survival (Hirsch et al., 2017). Conventional models utilize clinical tumor-node-metastasis (TNM) staging, vascular invasion, and other parameters to help predict LUAD prognosis (Jia et al., 2020). The efficacy of conventional models is still unsatisfying due to the heterogeneity of LUAD. There is a formidable barrier to fulfill the clinical promise of high-quality care and reduce LUAD burden (Hamann et al., 2018). As a result, it is urgent to develop more accurate biomarkers to predict prognosis of LUAD patients.

AU-rich elements (ARE) are crucial cis-acting sequences in the 3′ UTR, which mediate the recognition of a series of RNA-binding proteins (Khabar, 2010). Specifically, the forms of ARE sequences include U-rich or AU-rich sequences, repeats of overleaping pentamers of AUUUA and the nonamers UUAUUUAUU, and the latter two forms are recognized as the minimally functional ARE sequence (Khabar, 2010; Zubiaga, Belasco & Greenberg, 1995). The AU-rich mRNAs are a cluster of mRNA containing ARE in the 3′ untranslated regions (3′ UTR), accounting for 10–15% of all transcripts (Halees, El-Badrawi & Khabar, 2008). It is recognized that the length of 3′ UTR is a determinant factor in RNA stability. It appears that the 3′ UTR of ARE-mRNA is longer than non-ARE mRNAs such as those of housekeeping genes (Al-Zoghaibi et al., 2007; Mukherjee et al., 2009). Longer 3′ UTR tend to have a higher proportion of miRNA targets and the higher order of post-transcriptional regulation complexity (Jing et al., 2005). Binding the RNA-binding proteins or synergized by certain miRNA, ARE mediate rapid mRNA decay and thus affect translation of the ARE-mRNA (Khabar, 2010). The genes coding for ARE-mRNA include cytokines, growth factors and certain receptors such as VEGF, CCL2, EGF, EGFR et al., most of which play important roles in chronic inflammation and cancer (Khabar, 2010). Tumor necrosis factor (TNF-α) is an ARE-gene. It is recognized that TNF-α is a pro-inflammatory cytokine and plays an important role in triggering production of other inflammatory mediators including IL-8, IFN-γ, CXCL10 and so on (Kontoyiannis et al., 1999). Despite the notion of its name as tumor necrosis protein, TNF-α promotes initiation and development of multiple cancers including hepatocellular carcinoma, colorectal cancer, epithelial ovarian cancer and lung cancer et al. (Pikarsky et al., 2004; Popivanova et al., 2008; Kulbe et al., 2007; Zhao et al., 2018). COX-2 is another ARE-gene which catalyzes the key step in the prostaglandin production pathway. The increased stability and activity of COX-2 are associated with colon and other cancers, and contribute to cellular proliferation, resistance to apoptosis, angiogenesis and metastasis (Tsujii et al., 1998; Sawaoka et al., 1999; Li, Yang & Yan, 2002). Moreover, pro-inflammatory cytokines, many of which are coded by ARE-genes, such as IL-1, IL-6, are released from the tumor microenvironment favoring the initiation and progression of tumors (Khabar, 2010). Consequently, ARE-genes are intimately associated with tumor and have great potential to become new therapeutic targets of cancer.

Although there is increasing evidence suggesting that the ARE-genes play important roles in tumorigenesis and progress (Haghnegahdar et al., 2000). The prognostic value of ARE-genes on LUAD remains ambiguous. Here, we analyzed differentially expressed ARE-genes between LUAD and adjacent tissues. Then, by univariate, LASSO, multivariate Cox regression analyses, a prognostic genetic signature containing 12 genes was identified and was combined with clinicopathological features to establish a prognostic model. Kaplan–Meier curve, Harrell’s concordance index (C-index), calibration curves and decision curve analyses (DCA) were used to assess accuracy and reliability of the model. Furthermore, function enrichment analysis, immunity and tumor mutation burden (TMB) analyses were performed to further explore the underlying molecular mechanisms. GEO data were used for external validation. Finally, we explored the predicting role of AU-rich genes on prognosis and therapeutic effects of LUAD.

## Materials and Methods

### Data download

The mRNA expression profile, mutation data and clinicopathological information of LUAD were obtained from TCGA database (discovery cohort) (https://portal.gdc.cancer.gov/). To validate the accuracy of the prognostic model, an independent dataset was downloaded from GEO database (GSE13213) (Tomida et al., 2009) (validation cohort) (https://www.ncbi.nlm.nih.gov/geo/query/acc.cgi?acc=GSE13213). The TCGA dataset met the following criteria: (1) human LUAD and adjacent tissue; (2) integrated follow-up information; (3) the number of samples > 30. The genes coding for ARE (ARE-genes) were acquired from the human AU-rich element-containing mRNA database (ARED) (https://brp.kfshrc.edu.sa/ared) (Bakheet, Hitti & Khabar, 2018). Data were downloaded from the publicly available database. Hence it was not applicable for additional ethical approval.

### Identification of a prognostic genetic signature

First, we identified the differentially expressed genes (DEGs) between LUAD and adjacent samples in TCGA cohort using Wilcoxon test in R. —log_2_FC— > 1 and false discovery rate (FDR) < 0.05 were set as the cutoffs for the DEGs. Then the intersection of DEGs and AREs (DE-AREs) was visualized *via* a venn diagram and used to further analyze. Univariate Cox regression was performed and *P* < 0.01 was considered statistically significant. The DE-AREs which were significant in univariate Cox regression were further filtered by least absolute shrinkage and selector operation (LASSO) and multivariate Cox regression analyses (McEligot et al., 2020). Finally, a prognostic genetic signature was identified.

### Construction and validation of the AREs-based prognostic risk score model

According to the prognostic genetic signature, we calculated the risk scores of each patient in TCGA cohort based on a linear combination of the multivariate Cox regression coefficients (β) multiplied with its mRNA expression level. The risk score = (β_mRNA1_ * expression level of mRNA1) + (β_mRNA2_ * expression level of mRNA2) + (β_mRNA3_ * expression level of mRNA3) + ⋯ + (β_mRNAn_ * expression level of mRNAn) (Liu et al., 2019). The patients were divided into the high- and low-risk groups according to their risk scores. Kaplan–Meier plotter and C-index analysis were conducted to evaluate the model of the 12-gene signature. To elevate the accuracy of the predicting model, we performed univariate and multivariate Cox regression to screen the conventional clinical attributes including age, gender, stage, treatment type, primary site, and race. Finally, a nomogram consisting of the genetic signature and clinical attributes was constructed to predict the patients’ overall survival (OS). The performance of this prognostic nomogram was evaluated by Kaplan–Meier analysis, C-index, calibration curve and decision curve analyses(DCA) (Vickers & Elkin, 2006). To validate the reliability of the prognostic model, a GEO dataset (GSE13213) was used for the external validation.

### Expression analyses of prognostic genes by GEPIA and HPA

To detect the mRNA and protein expression level of prognostic genes, we utilized two online tools. GEPIA (Gene Expression Profiling Interactive Analysis) (http://gepia.cancer-pku.cn/) is a web-based tool to provide series of functions including differential expression analysis, profiling plotting, correlation analysis et al. based on TCGA and GTEx data (Tang et al., 2017). The human protein atlas (HPA) (https://www.proteinatlas.org/) is an online website which provides the expression information of a number of proteins in normal or pathologic tissues.

### Function enrichment analyses and tumor immunity analyses

In order to analyze gene enrichment difference between high- and low-risk groups, GO and KEGG pathway analyses were performed (GSEA: version: 4.0.3) and visualized by bioinformatics online tool (http://www.bioinformatics.com.cn/). GO enrichment analyses include biological processes (BP), cellular components (CC), molecular functions (MF). Via single sample Gene Set Enrichment Analysis (ssGSEA), each patient obtained a score according to 29 genesets related to immunity and all patients were separated to different subtypes. Stromal, immune and estimate scores were calculated with the ESTIMATE (estimation of stromal and immune cells in malignant tumor tissues using expression data) algorithm (Bi et al., 2020). Furthermore, we analyzed the correlation of risk scores and ImmuneScores, StromalScores, tumor purity, respectively and the survival curves of high, medium, and low immune subtypes were drawn. In addition, we also evaluated the infiltrating levels of various immune cells including B cells memory, T cells CD4 memory resting, T cells CD4 memory activated and T cells regulatory et al. Besides, the expression of a series of HLA-related genes, classical markers in chemotherapy-induced immune response and immune checkpoint genes between high- and low-risk groups were analyzed (Danilova et al., 2019).

### Gene mutation analyses

The landscape of gene mutation between high- and low-risk groups was analyzed using single nucleotide variation data in TCGA. By the Maftools package, Somatic mutation data were visualized based on the Mutation Annotation Format (MAF) file (Mayakonda et al., 2018). The top 20 genes with the highest mutation frequency in high- and low-risk groups were manifested in waterfall charts. The chip-square test was performed to compare the difference of total mutation and the top 3 genes mutation frequency between high- and low-risk groups. The TMB for each patient was calculated as follows: TMB = (total count of variants)/(the whole length of exons). Then, we compared the TMB of high-risk group with that of low-risk group.

### Statistical analysis

Statistical analysis was performed in R v.4.0.2 and SPSS. Unless otherwise stipulated, all statistical tests were two-sided, and *P* < 0.05 was considered statistically significant.

## Results

### Identification of differentially expressed ARE-genes

**Figure 1:**
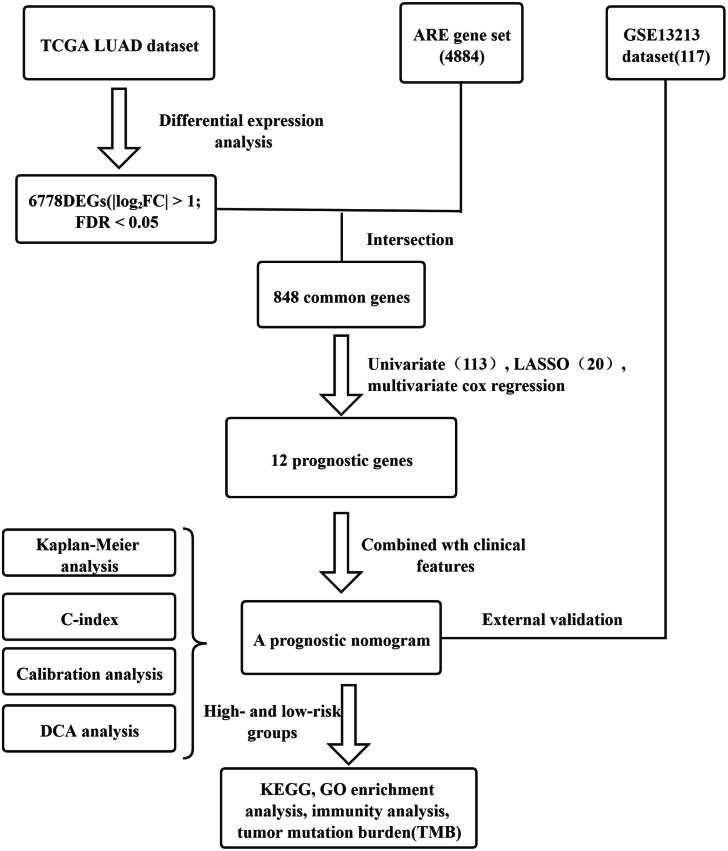
The flow chart showing the scheme of the study on the prognostic signatures for lung adenocarcinoma.

**Figure 2:**
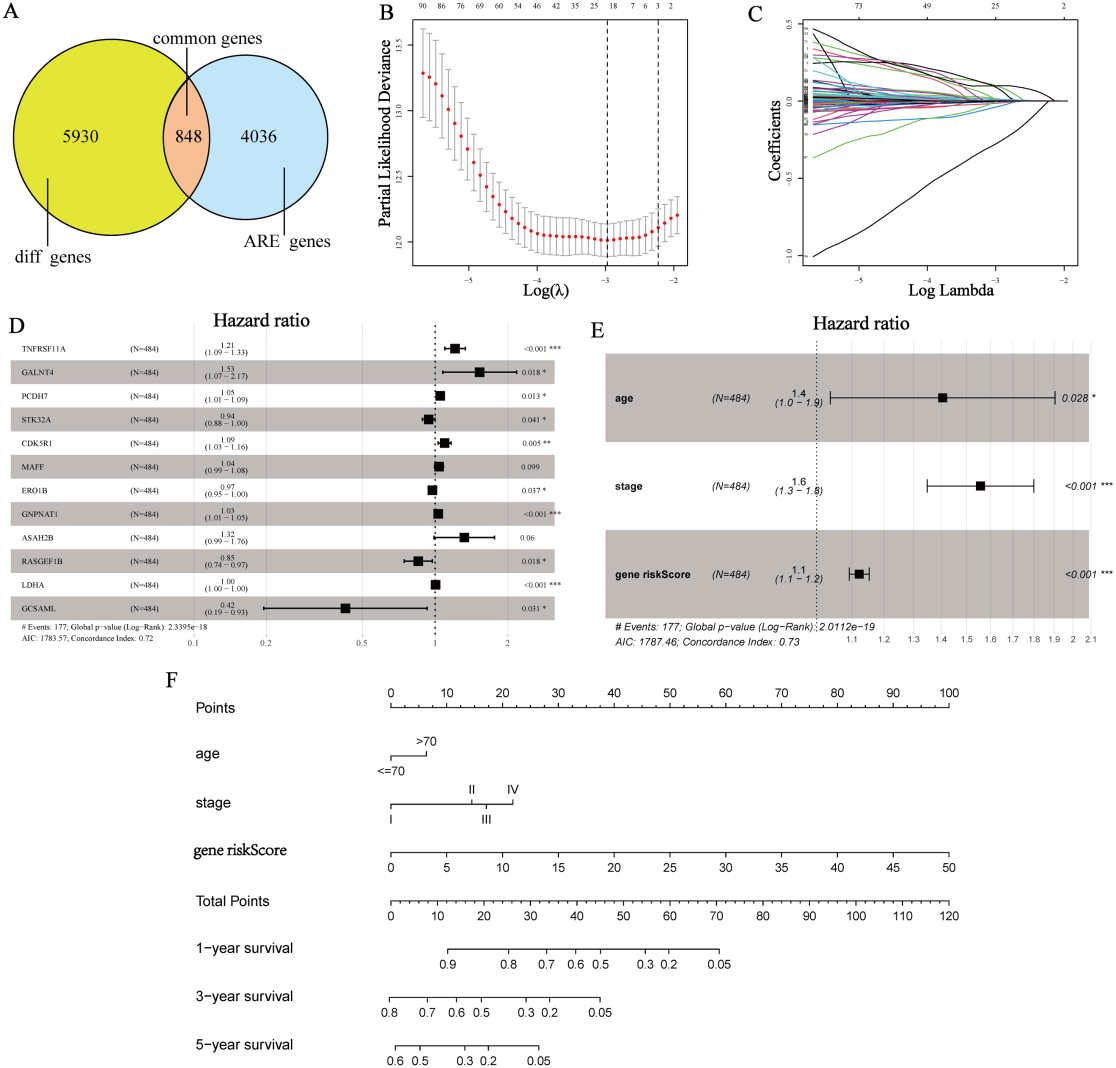
Construction of the AREs-based prognostic risk score model. (A) A total of 848 differentially expressed ARE-genes between LUAD and normal tissues. (B, C) Determination of the number of genes by the LASSO analysis. (D) Forest map of 12 prognostic genes by multivariate Cox regression analysis. (E) Forest map of clinicopathological attributes and gene riskScore by multivariate Cox regression analysis. (F) Prognostic nomogram to predict the survival of LUAD patients based on the TCGA discovery cohort.

This study was conducted according to the flow chart shown in Fig. 1. Details of the TCGA and GEO datasets in this study were shown in Table 1. We obtained 6778 DEGs between tumor and adjacent tissues of the TCGA dataset using Wilcoxon test by R (version 4.0.2). From the ARED, a total of 4884 ARE-genes were obtained. Intersecting the DEGs and ARE-genes, 848 common genes were screened out (Fig. 2A).

**Table 1:** Demographics and clinicopathologic characteristics of LUAD patients in the TCGA discovery cohort and GEO validation cohort.

**Variables**	**Discovery cohort**	**Validation cohort**
	**TCGA (484)**	**GSE13213 (117)**
**Gender**		
Male	221 (45.7)	60 (51.3)
Female	263 (54.3)	57 (48.7)
**Age at diagnosis**		
Mean (SD)	65.2 (10.06)	60.7 (10.17)
**Stage**		
I	263 (54.3)	79 (67.5)
II	117 (24.2)	13 (11.1)
III/IV	104 (21.5)	25 (21.4)
**Survival event**		
Alive	307 (63.4)	68 (58.1)
Dead	177 (36.6	49 (41.9)
**Median survival time**		
Time, Days (Range)	876.4 (4–7248)	1936.8 (186–3295)
**Treatment type**		
Chemotherapy	250 (51.7)	NA
Radiotherapy	234 (48.3)	NA
**Metastasis (Lymph nodes or distance)**		
No	202 (41.7)	87 (74.4)
Yes	174 (36)	30 (25.6)
NA	108 (22.3)	0 (0)

**Notes.**

SD, standard deviation.

### Identifying genes for the prognostic model

First, by univariate Cox regression analysis, 113 genes were filtered from the 848 common genes (*P* < 0.01). Then performing LASSO regression, the number of genes was further reduced to 20 (*λ* = −3) (Figs. 2B, 2C). After multivariate Cox regression, 12 genes were finally determined to construct the prognostic model. They were TNFRSF11A, GALNT4, PCDH7, STK32A, CDK5R1, MAFF, ERO1B, GNPNAT1, ASAH2B, RASGEF1B, LDHA, GCSAML. The hazard ratios of these genes were presented by a forest map (Fig. 2D). STK32A, ERO1B, RASGEF1B and GCSAML were significantly low hazardous genes and TNFRSF11A, GALNT4, PCDH7, CDK5R1, MAFF, GNPNAT1 and ASAH2B were high hazardous genes. Next, we detected the expression of these genes at mRNA and protein levels by GEPIA and HPA (Figs. S1, S2). The results suggested that only two genes (MAFF and RASGEF1B) out of the twelve prognostic genes were significantly down-regulated in LUAD and the others except for GCSAML were up-regulated at the transcription level. At the protein level, MAFF, GNPNAT1 and RASGEF1B expression were significantly down-regulated in LUAD and PCDH7, LDHA and GCSAML expression were up-regulated. STK32A, CDK5R1, ASAH2B and GALNT4 were not found in the HPA.

### Construction and validation the prognostic model

**Figure 3:**
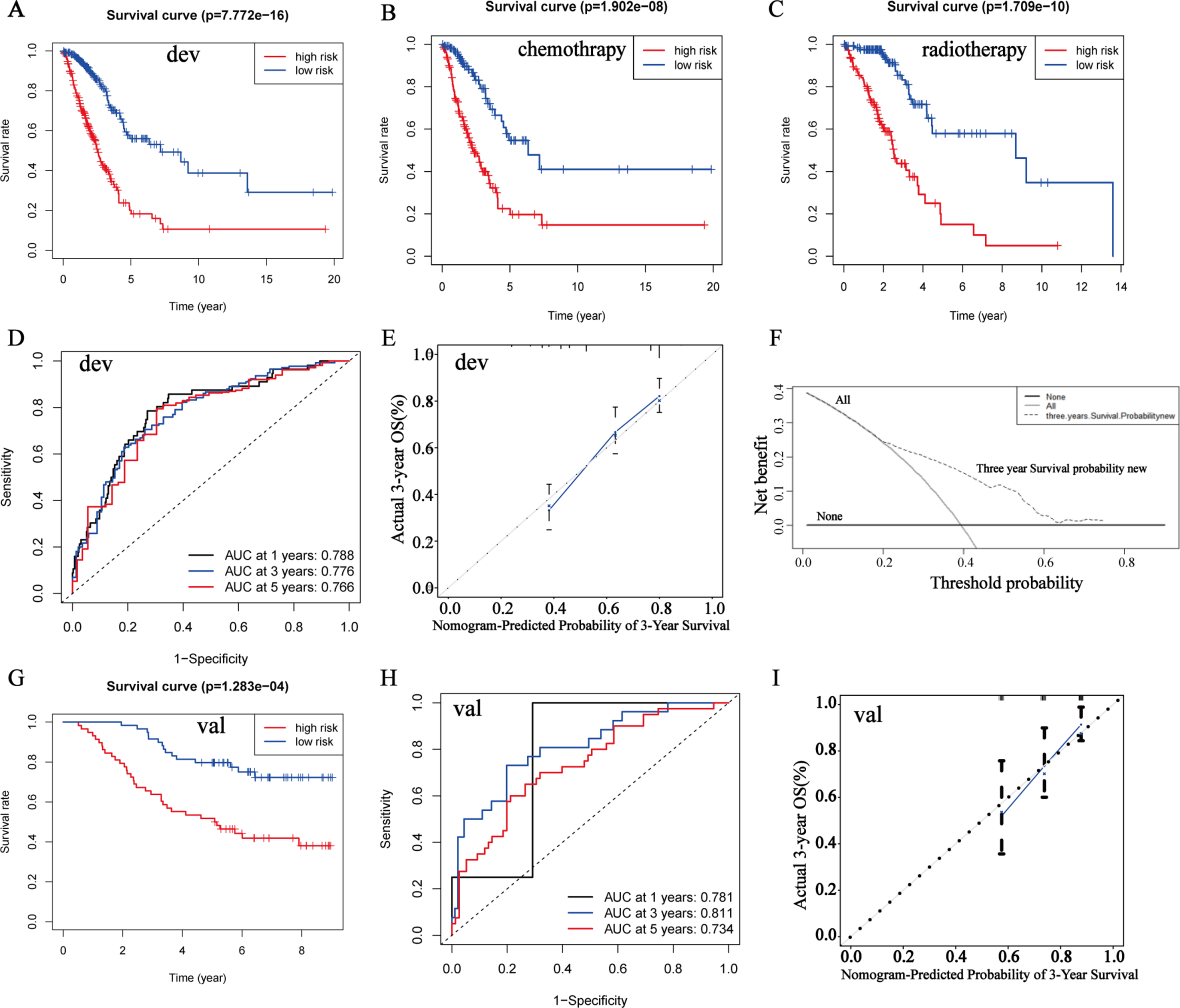
Kaplan–Meier analyses, C-index, calibration curves and DCA curves of the prognostic nomogram. dev: TCGA discovery cohort; val: GSE13213 validation cohort. (A) Kaplan–Meier analyses of OS between the high- and low-risk groups in TCGA discovery cohort. (B, C) Survival analyses of high- and low-risk groups receiving chemotherapy and radiotherapy, respectively. (D) 1, 3, 5-year ROC curves of the high- and low-risk groups in TCGA discovery cohort. (E) Calibration curves of the high- and low-risk groups in TCGA discovery cohort. The *Y*-axis represents actual survival, and the *X*-axis represents nomogram-predicted survival. (F) DCA curves of 3-year survival probability of the prognostic nomogram in TCGA discovery cohort. (G, H, I) Kaplan–Meier analyses, C-index, calibration curves of GSE13213 validation cohort.

The model of the 12-genes signature showed a strong predicting ability for OS and 1, 3, 5-year AUC of receiver operating characteristic (ROC) curve were 0.789, 0.752, 0.723 respectively (Figs. S3A, S3B). The risk score analyses of patients’ survival status, survival time and gene expression were showed in Fig. S3C. To elevate the accuracy of the prognostic model, several conventional clinical attributes including age, gender, stage, treatment type, primary site and race were screened by univariate and multivariate Cox regression analyses. The result of univariate Cox regression demonstrated that only age and stage had statistical significance (age: HR 1.44, 95% CI [1.06–1.95] *P* = 0.018; stage II: HR 2.34, 95% CI [1.62–3.38], *P* < 0.001, stage III: HR 3.27, 95% CI [2.23–4.81], *P* < 0.001; stage IV: 3.58, 95% CI [2.06–6.20], *P* < 0.001) (Table 2). The other attributes such as gender, treatment type, primary site and race have no significant difference. Multivariate Cox regression also validated that age, stage and gene riskScore were independent prognostic factors (age: HR 1.4, 95% CI [1.0–1.9], *P* = 0.028; stage: HR 1.6, 95% CI [1.3–1.8], *P* <  0.001; gene riskScore: HR 1.1, 95% CI [1.1–1.2], *P* < 0.001) (Fig. 2E). Finally, age and stage were added to the model and a prognostic nomogram was drawn (Fig. 2F). The nomogram showed that gene riskScore played the most important role in the LUAD prognosis followed by stage and age. In the nomogram, any category of the variables was matched with a score. By collecting the variables and calculating the total score of the patient, clinicians could speculate 1-/3-/5-year survival probability *via* drawing a line straight down to survival probability scale from the total Points scale. As a result, clinicians could more easily evaluate survival rate of LUAD patients by the nomogram. According to the risk scores’ median, the patients were divided into high- and low-risk groups. By Kaplan–Meier plotter, it suggested that the high-risk group had a significantly worse prognosis than the low-risk group (*P* = 7.772e−16) (Fig. 3A). To further analyze the correlation of patients’ risk scores and chemotherapy and radiotherapy resistance, we compared the survival curves of high- and low-risk groups which accepted chemotherapy and radiotherapy, respectively. The results consistently manifested that the high-risk group had worse prognosis (Figs. 3B, 3C). The C-index was used to evaluate the predicting ability of the prognostic model. 1, 3, 5-year of AUC were 0.788, 0.776, 0.766, which suggested that the model had a high discrimination (Fig. 3D). To detect the calibration of the model, the 3-year calibration curve was drawn and reflected a robust consistency (Fig. 3E). In addition, we also plotted a DCA to ascertain the clinical usefulness (Fig. 3F). The results demonstrated that the prognostic model had high net benefit with abundant ranges of threshold probabilities, which indicated that the model possessed good clinical applicability in predicting 3-year survival rate. To perform an external validation, the GSE13213 dataset was downloaded and utilized to perform the survival analysis, C-index, calibration curve (Figs. 3G–3I). The results manifested a good concordance with the discovery cohort.

**Table 2:** Univariate Cox regression analysis of overall survival (OS).

**Variables**	**n**	**Overall survival (OS)**	
		**HR (95% CI)**	***P*** **Value**
**Age**	484	
≤70	325	1	–
>70	159	1.44 (1.06–1.95)	0.018
**Gender**	484	
Female	263	1	–
Male	221	1.08 (0.80–1.45)	0.623
**Stage**	484	
I	263	1	–
II	117	2.34 (1.62–3.38)	<0.001
III	79	3.27 (2.23–4.81)	<0.001
IV	25	3.58 (2.06–6.20)	<0.001
**Treatment type**	466	
Chemotherapy	239	1	–
Radiotherapy	227	0.79 (0.59–1.08)	0.14
**Primary site**	466	
Lower lobe	163	1	–
Middle lobe	20	0.99 (0.40–2.46)	0.978
Upper lobe	283	0.83 (0.60–1.13)	0.228
**Race**	432	
People of European	382	1	–
African and Latin-American descent	50	0.71 (0.42–1.19)	0.195

**Notes.**

Abbreviations CI confidence interval HR hazard ratio

### Function enrichment analyses

**Figure 4:**
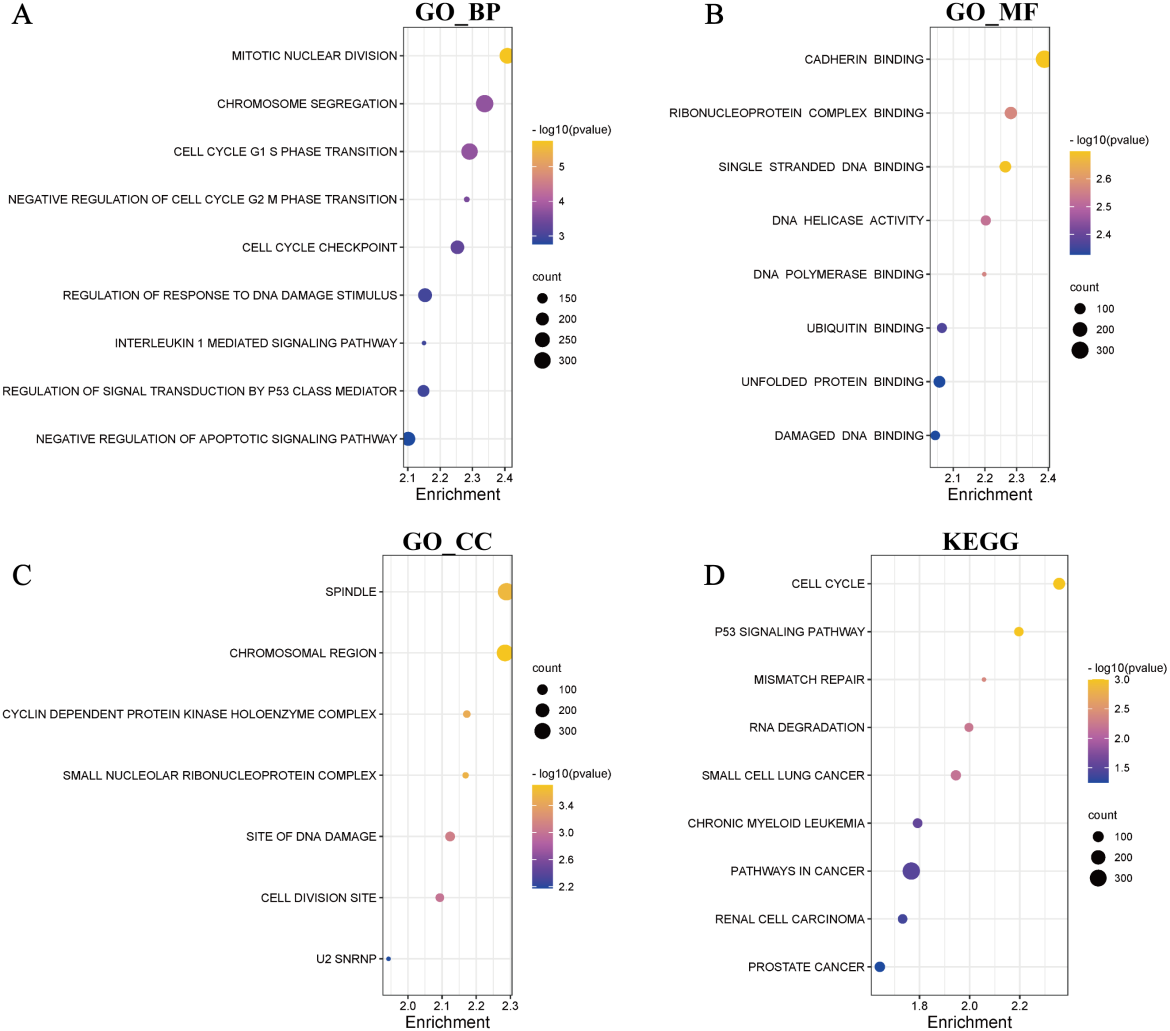
Function enrichment analyses. GO enrichment analyses, including BP (biological process) (A), MF (molecular function) (B), CC (cellular component) (C). (D) KEGG enrichment analyses.

To explore the underlying molecular mechanism of difference of OS between the high- and low-risk groups, we performed KEGG and GO enrichment analysis (Figs. 4A–4D). The results of KEGG showed that multiple carcinogenic pathways were enriched including small cell lung cancer, chronic myeloid leukemia, renal cell carcinoma and prostate cancer. In addition, cell cycle and P53 signaling pathway also were enriched in the high-risk group. Interestingly, the results of GO enrichment analysis were significantly correlated with cell division, cell cycle checkpoint, DNA damage response and cell apoptosis whether in biological processes (BP), cell components (CC) or molecular functions (MF). The inflammation pathway also occupied the certain position in the results.

### Tumor immunity analyses

**Figure 5:**
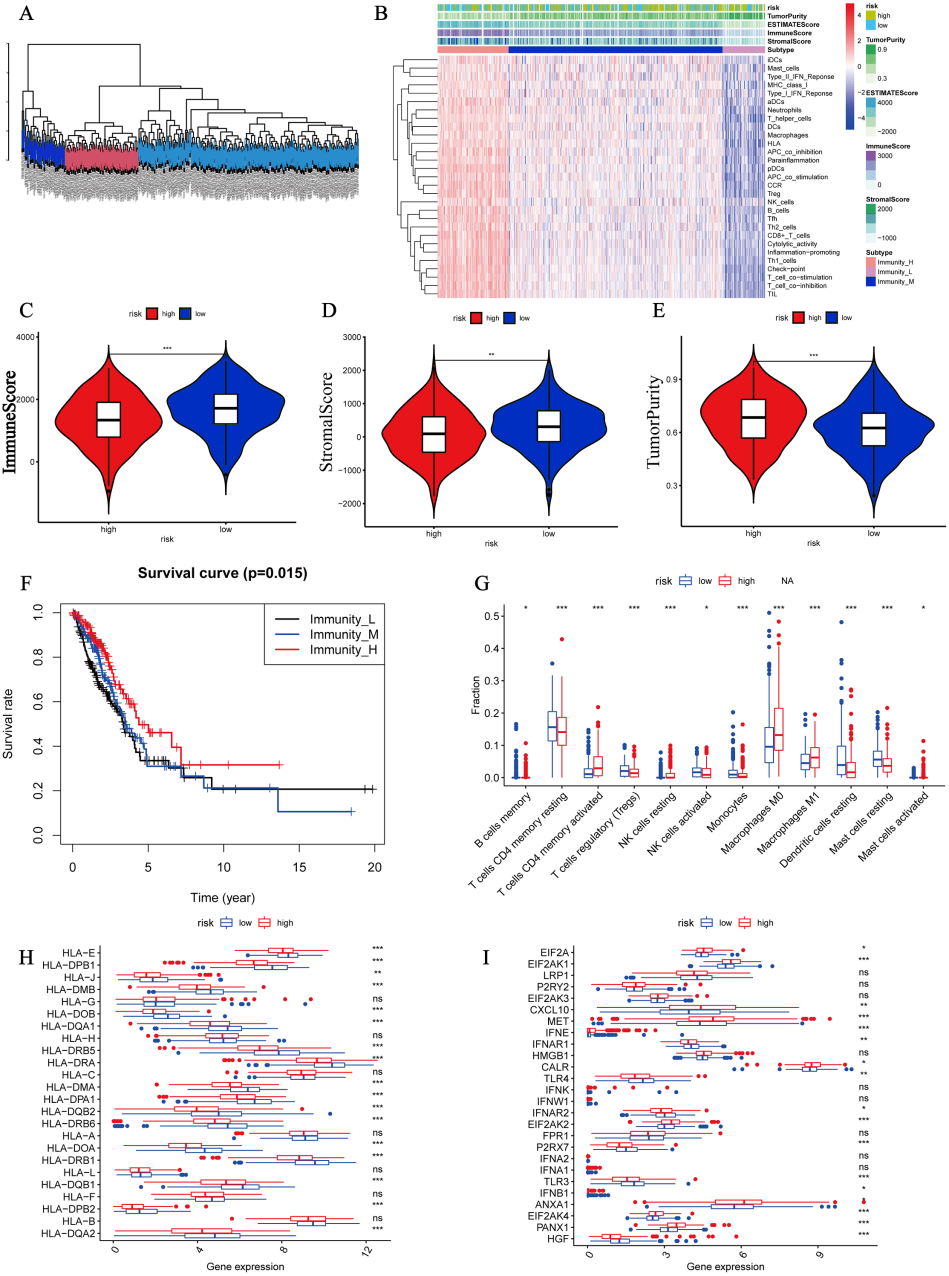
Tumor immunity analyses. (A) The TCGA LUAD patients were divided into three subtypes by single sample Gene Set Enrichment Analysis (ssGSEA). (B) A heatmap showing the correlation of risk score, ImmuneScore, StromalScore, tumor purity, EstimateScore and various immune cells and immune pathways. (C) The correlation of risk score and immuneScore. (D) The correlation of risk score and stromalScore. (E) The correlation of risk score and tumor purity. (F) Kaplan–Meier curves of high, medium and low immuneScore group. Immunity_L: low immuneScore group; Immunity_M: medium immuneScore group; Immunity_H: high immuneScore group. (G) The infiltrating levels of various immune cells between the high- and low-risk groups. (H) The differential mRNA expression of HLA-related molecules between the high- and low-risk groups. (I) The differential expression of 26 genes associated with chemotherapy-induced immune response between the high- and low-risk groups. * *P* < 0.05, ** *P* < 0.01, *** *P* < 0.001.

According to the ssGSEA, the TCGA LUAD patients were divided into three subtypes (Fig. 5A). We also drew a heatmap to show the correlation of risk score, ImmuneScore, StromalScore, tumor purity, EstimateScore, various immune cells and immune pathways (Fig. 5B). Further, we found that compared to the high-risk group, the low-risk group had higher ImmuneScore (*P* < 0.001) and StromalScore (*P* < 0.01) (Figs. 5C, 5D). Hence, the low-risk group was lower on the aspect of tumor purity (*P* < 0.001) (Fig. 5E). The Kaplan–Meier plotter revealed that survival rates were increased with immune scores (Fig. 5F). The results of immune cell filtrating suggested that B cells memory, T cell CD4 memory resting, T cell regulatory, NK cell activated, monocytes, dendritic cells resting, mast cells resting were decreased and T cells CD4 memory activated, NK cells resting, macrophages M0, macrophages M1, mast cells activated were increased in the high-risk group (Fig. 5G).

**Figure 6:**
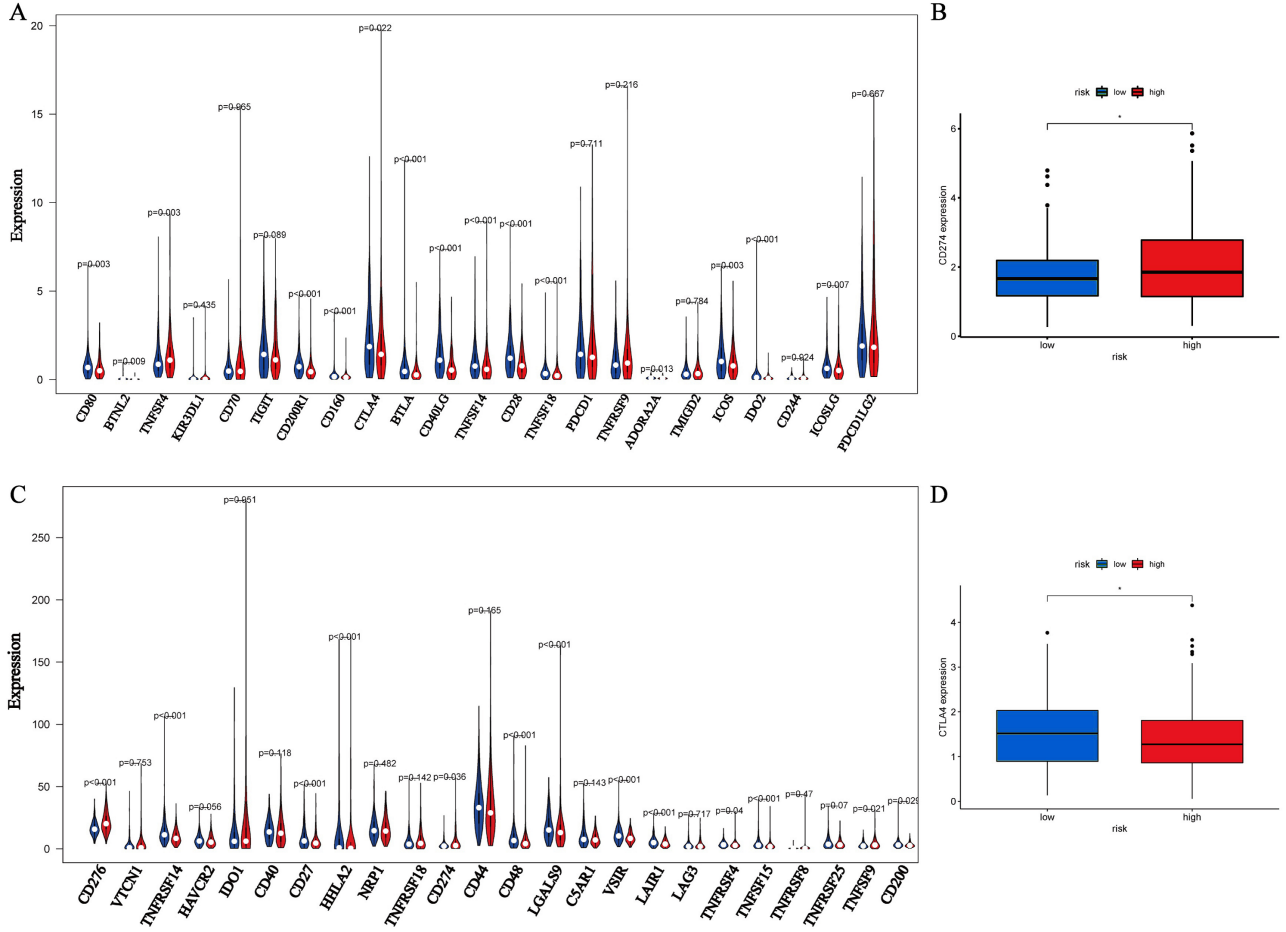
The expression analyses of immune checkpoint genes. (A, C) The differential expression of 47 immune checkpoint genes between the high- and low-risk groups. (B) The expression of PD-L1 between the high- and low-risk groups. (D) The expression of CTLA4 between the high- and low-risk groups.

As for HLA-related genes expression, it was common that most important HLA-related genes were significantly down-regulated in the high-risk group (Fig. 5H). The expression of 26 genes associated with chemotherapy-induced immune response was showed as Fig. 5I. 17 (65.4%) genes were significantly differentially expressed, which suggested that different risk groups had different responses to chemotherapy. On the aspect of immune checkpoint genes, 28 (59.6%) genes were differentially expressed (Figs. 6A, 6C) and notably, CD274 and CTLA4 had opposite expression. The high-risk group had higher CD274 expression but lower CTLA4 expression (Figs. 6B, 6D), which may imply that the high-risk group were more appropriate to anti-PD-L1 therapy rather than anti-CTLA4 therapy.

### Gene mutation analyses

By analyzing the landscape of gene mutation between high- and low-risk groups, two waterfall diagrams were plotted to show the top 20 genes with the highest mutation frequency (Figs. S4A). There was slightly higher mutation frequency in high-risk group than that in low-risk group (91.1% *vs* 86.19%). However, the total and top 3 genes mutation (TP53, TTN, MUC16) had no significant difference between high- and low-risk groups (all *P* value > 0.05) (Table S1). To investigate whether TMB was associated with risk score, we compared the TMB between high- and low-risk groups. There was no significant difference between the two groups (*P* = 0.32) (Fig. S4C).

## Discussion

Lung cancer remains a major challenge for public health worldwide (Bade & Cruz, 2020). As the main histological subtype, LUAD possesses complex carcinogenic mechanisms and obvious tumor heterogeneity. Although great progress has been made in diagnosis and treatment of LUAD, the prognosis for LUAD patients remains unsatisfying, with a 5-year survival rate ranging from ∼10% to ∼15%. Early metastasis, disease relapse, and drug resistance are common causes of mortality in LUAD patients (Wu et al., 2012). So it is greatly important to develop more effective biomarkers to predict prognosis of LUAD patients.

ARE, as an important component of post-transcriptional control, are closely correlated with mRNA decay (Yang et al., 2003). ARE-mediated changes are usually transient responses physiologically. Once the process of the ARE-mRNAs degradation is broken, it will cause prolonged responses that subsequently may lead to undesirable, such as diseased states including chronic inflammatory diseases and cancer (Khabar, 2010). For example, uPA, as an ARE-gene, is up-regulated in multiple cancers and stimulates angiogenesis to provide abundant nutrients, oxygen for tumor cells (Andreasen, Egelund & Petersen, 2000). Consequently, the ARE-genes are a cluster of genes with great potential prognostic value in tumors. The value and novelty of this study is to indicate, for the first time by bioinformatic analysis, that the prognostic value of ARE-genes in LUAD and establish a nomogram to visualize the results.

In this study, we established a novel twelve-gene signature base on ARE-genes including TNFRSF11A, GALNT4, PCDH7, STK32A, CDK5R1, MAFF, ERO1B, GNPNAT1, ASAH2B, RASGEF1B, LDHA, GCSAML. Combined with age and stage, the prognostic model had good robustness and could accurately distinguish high- and low-risk patients (1, 3, 5-year ROC: 0.788, 0.776, 0.766). Previous studies had constructed several prognostic models of LUAD by other gene sets. Song et al. constructed a LUAD prognostic model consisting of immune-related genes and clinical factors (1, 3, 5-year ROC: 0.718, 0.668, 0.652) (Song et al., 2020). Wang et al. utilized TCGA data to establish an epigenetic signature-based model for LUAD patients’ prognosis (1, 3, 5-year ROC: 0.759, 0.747, 0.757) (Wang et al., 2020). Although there are numbers of prognostic models based on different gene sets, they still exist many problems, such as low accuracy, too much identified genes and too expensive testing fee et al. This is why researchers constantly try to construct new prognostic models. The genetic signature of 12 genes is acceptable in clinical practice and the accuracy is higher than most counterparts. The results of multivariate Cox regression analysis showed that TNFRSF11A, GALNT4, PCDH7, CDK5R1, MAFF, GNPNAT1, ASAH2B, LDHA were negative prognostic factors and STK32A, ERO1B, RASGEF1B, GCSAML were opposite. GCSAML, GALNT4 were the most significantly favorable and hazardous prognostic factors, respectively (GCSAML HR 0.42, 95% CI [0.19–0.93]; *P* value 0.03; GALNT4 HR 1.53 95% CI [1.07–2.17]; *P* value 0.02). GCSAML encodes a protein thought to be a signaling molecule associated with germinal centers, the sites of proliferation and differentiation of mature B lymphocytes (https://www.ncbi.nlm.nih.gov/gene/148823). GALNT4 (Polypeptide N-acetylgalactosaminyltransferase4) participates in initiation and progression of various cancers including colon cancer, non-small cell lung cancer, hepatocellular carcinoma and prostate cancer. Numerous miRNAs target GALNT4 to suppress the tumors, such as miR-4262, miR-506-3p, miR365b (Qu, Qu & Zhou, 2017; Xing et al., 2020; Hu et al., 2019; Liu et al., 2017). Consequently, designing novel drugs targeting the oncogenic ARE sequences specifically may be a new idea in the treatment of tumor. In addition, the function and prognostic values of the rest genes have been also studied in other tumors. miR-3150b-3p directly targets TNFRSF11a to inactivate the p38 MAPK signaling pathway and thus inhibits proliferation and metastasis of cervical cancer (Yu, Wang & Li, 2020). In colon cancer, oncogenic lncRNA LNAPPCC promotes metastasis and recurrence and contributes to bad prognosis *via* forming a positive feedback loop with PCDH7 (Li et al., 2020). Moreover, it has been verified that elevated expression of CDK5R1, GNPNAT1, LDHA is tightly associated with worse prognosis in diverse cancers including hepatocellular carcinoma, oral squamous cell carcinoma et al. (Zeng et al., 2021; Zheng et al., 2020; Cai et al., 2019). These results of the studies are consistent with our findings. As structurally conserved sequence in mRNA, ARE ubiquitously mediate the degradation of mRNA in mammalian cells (Otsuka et al., 2019; Chen & Shyu, 1995). Consequently, it is promising for our findings to apply in other cancers.

To explore the underlying molecular mechanism of different prognoses between high- and low-risk groups, function enrichment analyses and tumor immunity analyses were performed. DEGs of the high- and low-risk groups mainly pointed to cell cycle, DNA damage response, inflammatory response and cancer. The tumor cell proliferation and chemotherapy resistance of tumor cells were strongly associated with cell cycle and DNA damage response (Wu et al., 2019; He et al., 2017). Considerable oncogenic signaling pathways are linked to deregulation of cell cycle including PI3K/AKT signaling pathway, inactivation of P53-DREAM pathway et al. (Sizek et al., 2019; Engel, 2018). It has been reported that P53 expression can be altered by ARE. P53 is a RNA binding protein and contains ARE in the 3′ UTR of its mRNA. More importantly, P53 can regulate its own expression through the binding to ARE sequences of P53-mRNA *via* translation-independent and translation-dependent polysome-associated pathways (Derech-Haim et al., 2020). As a vital onco-suppressor, change of stability of P53 Mrna by ARE may contribute to the difference of DNA damage response, which can further contribute to initiation, proliferation and chemotherapy of tumor cells (Hong et al., 2014). Sooncheol et al. have reported that p38 MAPK-MK2 signaling pathway stabilizes ARE mRNAs by phosphorylation and inactivation of Tristetraprolin in G0 phase, which permits expression of ARE mRNAs that promote chemoresistance (Lee et al., 2020). Targeting ARE-genes, new cell cycle inhibitors and inducers of DNA damage response may be developed. A growing body of evidence manifests that tumor microenvironment plays an important role on forming malignant phenotype of various tumors. The main components of tumor microenvironment are resident stromal cells and recruited immune cells (Bi et al., 2020). The correlation between stromal and immune scores and risk scores showed that the high-risk group had lower stromal and immune scores, which meant it had more tumor components and higher tumor purity. The survival analyses of different immune scores also showed that the survival rates decreased with the decreasing of immune scores, which indicated that induction of immune infiltration in high-risk group may increase the survival rate of LUAD patients. On the aspect of immune cells, B cells memory, T cell CD4 memory resting, T cell regulatory, NK cell activated, monocytes, dendritic cells resting, mast cells resting were decreased and T cells CD4 memory activated, NK cells resting, macrophages M0, macrophages M1, mast cells activated were increased in high-risk group. Immune cell infiltration is a considerable complex process and the anti- or pro-tumor effects of numerous immune cells depend on tumor types and stage. For example, tumor infiltrating dendritic cells can be immunogenic or tolerogenic dependent upon environment signals. Dendritic cells are usually tumor suppressive in early stages and become tumor promoting as the tumor progresses (Hinshaw & Shevde, 2019). Up to now, macrophage M1 is ubiquitously thought to be tumor promoting and macrophage M2 is the opposite (Yang & Zhang, 2017). The result of macrophage M1 infiltrating is consistent with the present researches, which indirectly manifests macrophage M1 plays an unfavorable role on LUAD prognosis. B cells, NK cell activated and dendritic cells resting were increasingly recruited in the low-risk group. B cells and dendritic cells have the ability of presenting tumor antigens and NK cell can kill tumor cells directly. They are important members of innate immunity and adaptive immunity and stunt the tumorigenesis and development (Hinshaw & Shevde, 2019; Terrén et al., 2019; Tokunaga et al., 2019). Many ARE-genes are responsible for coding cytokines, growth factors. It has been reported that the mouse model with ARE deleted IFN-γ displays increased numbers of plasmacytoid dendritic cells (pDCs) in bone marrow and spleen (Hodge et al., 2014). ARE not only affect mRNA stability but also their translational efficiency. In M0 macrophages, TNF- α mRNA is inhibited translationally but promoted upon cell activation (Zhang et al., 2002). As a result, ARE may regulate development, migration, differentiation and activation of various immune cells *via* its function of mRNA degradation and translational efficiency.

In addition, various HLA-related genes expression were significantly different in the high- and low-risk groups. In total, the low-risk group had higher HLA-related genes expression. HLA molecules play an important role on initiation and metastasis of tumors. HLA class I molecules on tumor cell surface are responsible for recognition by T cell and NK cell. Tumors can escape T cell response by losing HLA class I molecules. Compared to primary tumor, MHC class I phenotype of metastatic colonies can be highly diverse and is not necessarily the same as that of the primary tumor (Garrido & Aptsiauri, 2019). HLA class I alterations are an important factor determining clinical response to immunotherapy (Garrido, 2019). So the effects of immunotherapy maybe improved successfully by up–regulating HLA class I expression in high-risk group. Because many HLA-related genes contain ARE and complex interaction of genes, the alteration of HLA expression is reasonable. The survival analyses of chemotherapy and radiotherapy group showed that high-risk group had worse prognosis. The expression of chemotherapy-induced immune response and immune checkpoint-related genes also suggested the significant difference between high- and low-risk groups, which implied the potential reasons for the difference of therapies. CD274, namely PD-L1, was highly expressed in the high-risk group but another famous immunotherapy target, CTLA4 was down-regulated in high-risk group, which implied that the LUAD patients with high risk scores may be more appropriate for anti-PD-L1 therapy rather than anti-CTLA4 therapy. It has been reported that RAS signaling can up-regulate tumor cell PD-L1 expression through increasing PD-L1 mRNA stability *via* modulation of the AU-rich element-binding protein tristetraprolin (Coelho et al., 2017). In the future, drugs targeting ARE-sequences of specific DNA or RNA may be developed to reverse the high-risk patients’ therapeutic effects on immunotherapy, chemotherapy or radiotherapy and expand the therapeutic thoughts of tumors.

TMB is used to represent the number of somatic mutations per DNA megabase and has been an independent predictor of immunotherapy in many tumors. It has been reported that patients with high TMB had higher response rate and better prognosis than the patients with low TMB when receiving PD-1 blockade (Carbone et al., 2017). However, TMB between the high- and low-risk groups had no significant difference. Moreover, the total mutation frequency and the top 3 genes with the highest mutation frequency (TP53, TTN, MUC16) all had no significant difference in the two groups, which may indicate that the prognostic difference between high- and low-risk groups was not caused by gene mutation. The results are coincident with the classical function of ARE. We speculate that the altered expression of key genes mediated by ARE results in the survival difference.

However, the study still has some limitations. First, the detection of these genes is at the RNA level, so it is expensive and difficult to apply in the clinical setting. Second, the data of TCGA and GEO databases are mainly collected from people of European, African and Latin-American descent. When applied on other ethnicities, the conclusion may be a little different. Finally, all mechanical analyses in our study were descriptive, the underlying mechanism of the twelve genes remains to be clarified by further functional experiments.

## Conclusion

ARE-genes can reliably predict OS in LUAD and indicate the effects of chemotherapy and radiotherapy for LUAD patients. We hope our study may provide new targets for LUAD treatment.

##  Supplemental Information

10.7717/peerj.12275/supp-1 Supplemental Information 1 The mRNA expression of the 12 prognostic genes by GEPIA

10.7717/peerj.12275/supp-2 Supplemental Information 2 The representative protein expression of the 12 genes in LUAD and normal tissue

10.7717/peerj.12275/supp-3 Supplemental Information 3 The Kaplan–Meier analysis, C-index and risk score analyses of the genetic model (A) Kaplan–Meier curve of the high- and low-risk groups stratified only by the 12 prognostic genes. (B) 1, 3, 5-year ROC curves of the pure genetic model. (C) Risk score analyse s of the pure genetic model in TCGA discovery cohort. Upper panel: risk score curve of the nomogram. Middle panel: patient survival status and time distributed by risk score. Bottom panel: the heatmap of twelve genes in LUAD samples.

10.7717/peerj.12275/supp-4 Supplemental Information 4 Tumor mutation burden analyses of the high- and low-risk groups The waterfall charts of the top 20 mutant genes in low-risk group (A) and high-risk group (B). (C) The TMB between the high- and low-risk groups.

10.7717/peerj.12275/supp-5 Supplemental Information 5 Chi-square test for the total mutation frequency and the top 3 genes (TP53, TTN, MUC16) with the highest mutation frequency

## Additional Information and Declarations

### Data Availability

The following information was supplied regarding data availability: All data used are available at the TCGA, GEO and ARED databases. The discovery cohort data is available at TCGA (https://portal.gdc.cancer.gov/) (filter criteria: bronchus and lung, TCGA, TCGA-LUAD, transcriptome profiling, Gene Expression Quantification, HTSeq-FPKM). The validation cohort data is available at GEO: GSE13213. The genes coding for AU-rich elements (ARE-genes) are available at the human AU-rich element-containing mRNA database (ARED) (https://brp.kfshrc.edu.sa/ared).
