# Supplementary figures and images for: Prognostic model of AU-rich genes predicting the prognosis of lung adenocarcinoma
Source: PeerJ. 2021 Oct 8;9:e12275. doi: 10.7717/peerj.12275 (PMC8504460; doi:10.7717/peerj.12275)

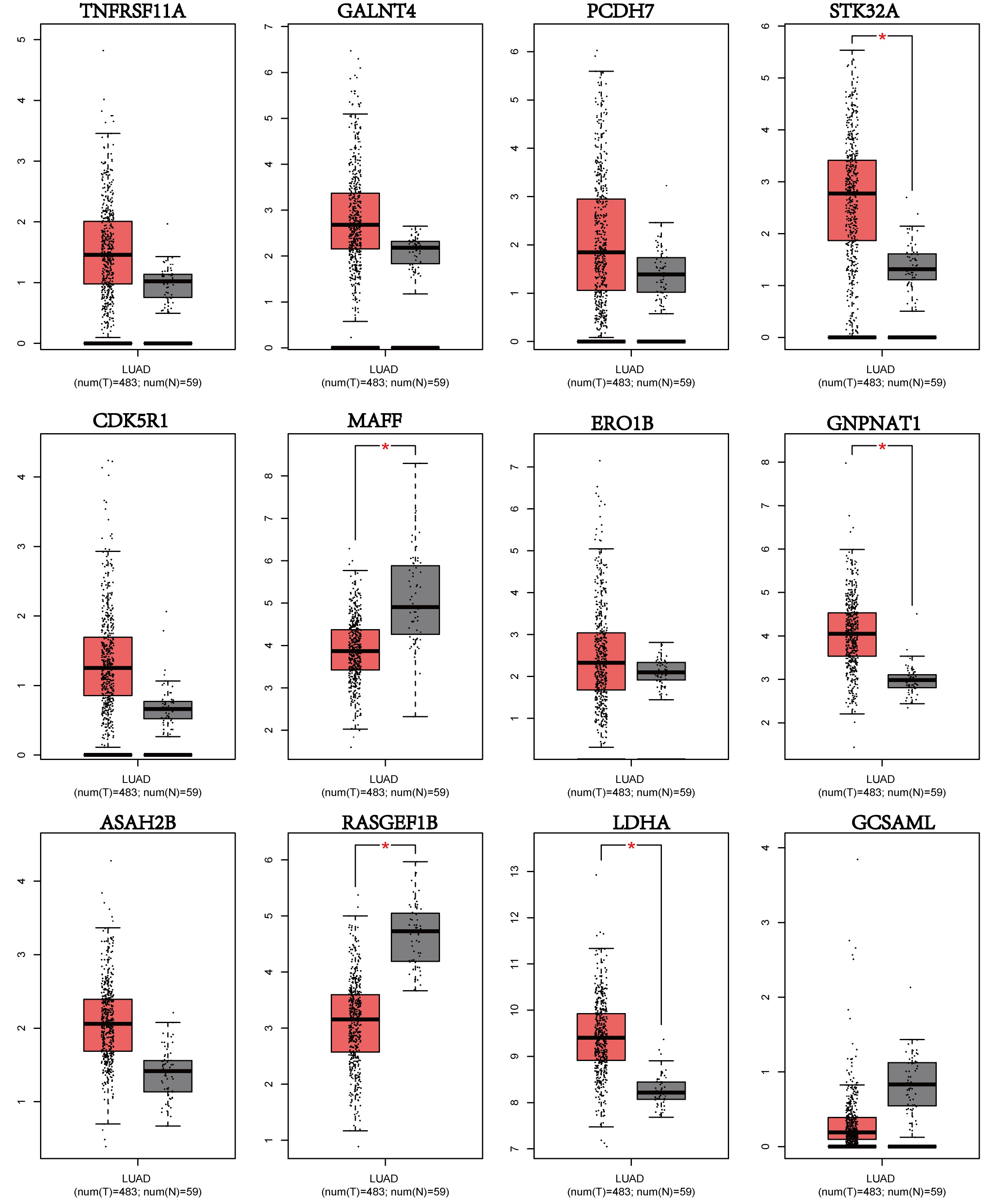

Supplement: Supplemental Information 1 [file peerj-09-12275-s001.png]

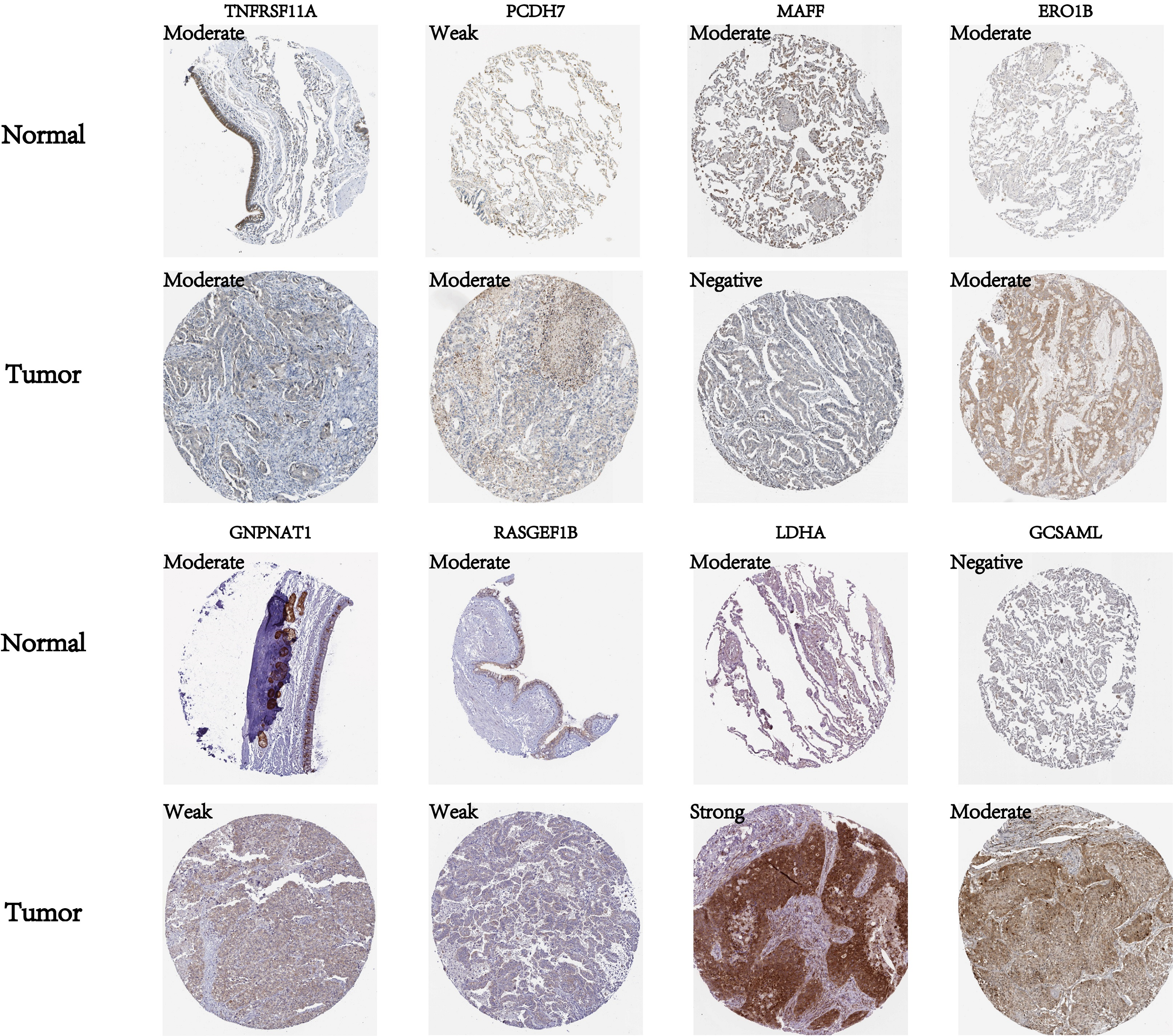

Supplement: Supplemental Information 2 [file peerj-09-12275-s002.png]

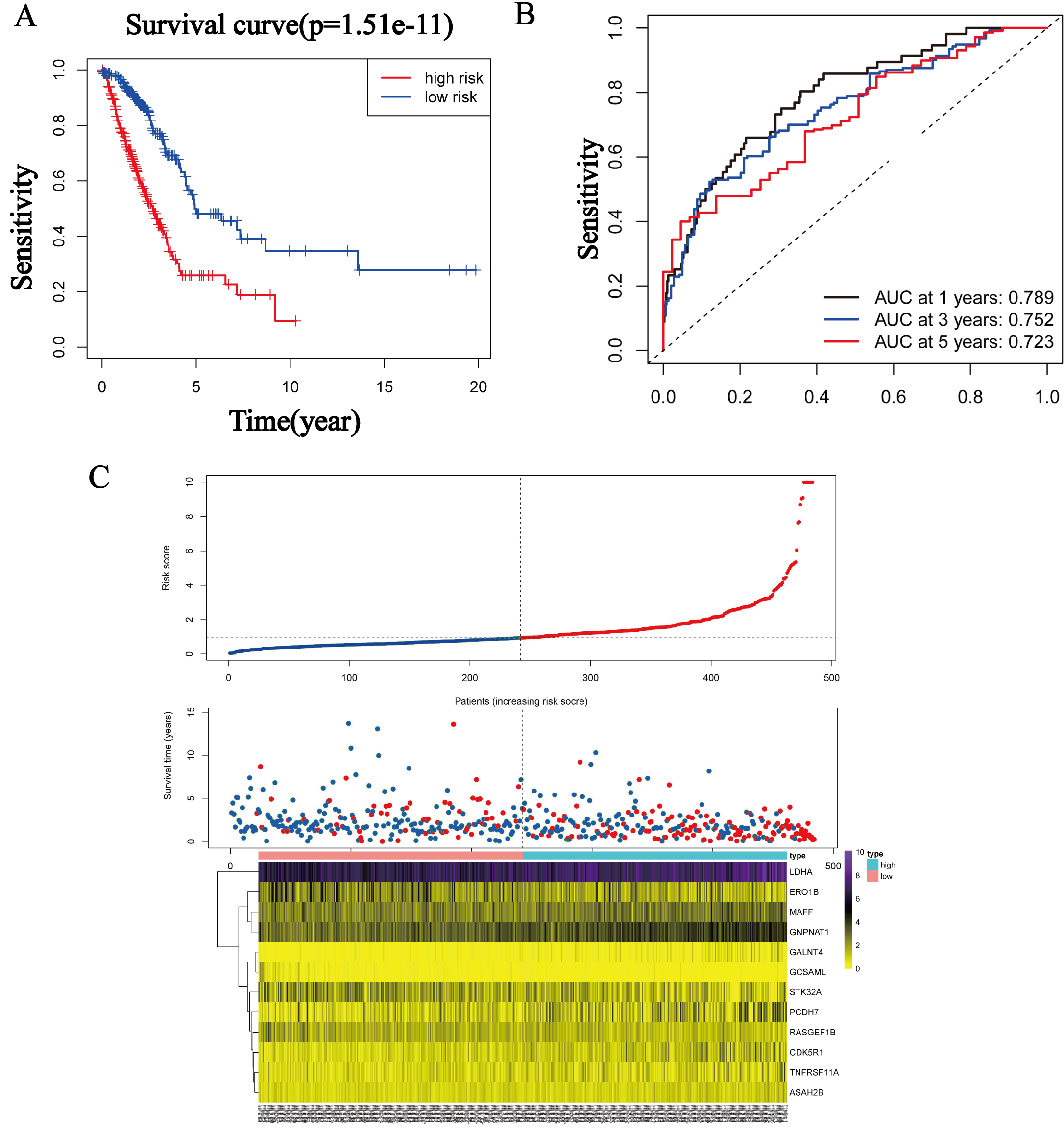

Supplement: Supplemental Information 3 — (A) Kaplan–Meier curve of the high- and low-risk groups stratified only by the 12 prognostic genes. (B) 1, 3, 5-year ROC curves of the pure genetic model. (C) Risk score analyse s of the pure genetic model in TCGA discovery cohort. Upper panel: risk score curve of the nomogram. Middle panel: patient survival status and time distributed by risk score. Bottom panel: the heatmap of twelve genes in LUAD samples. [file peerj-09-12275-s003.png]

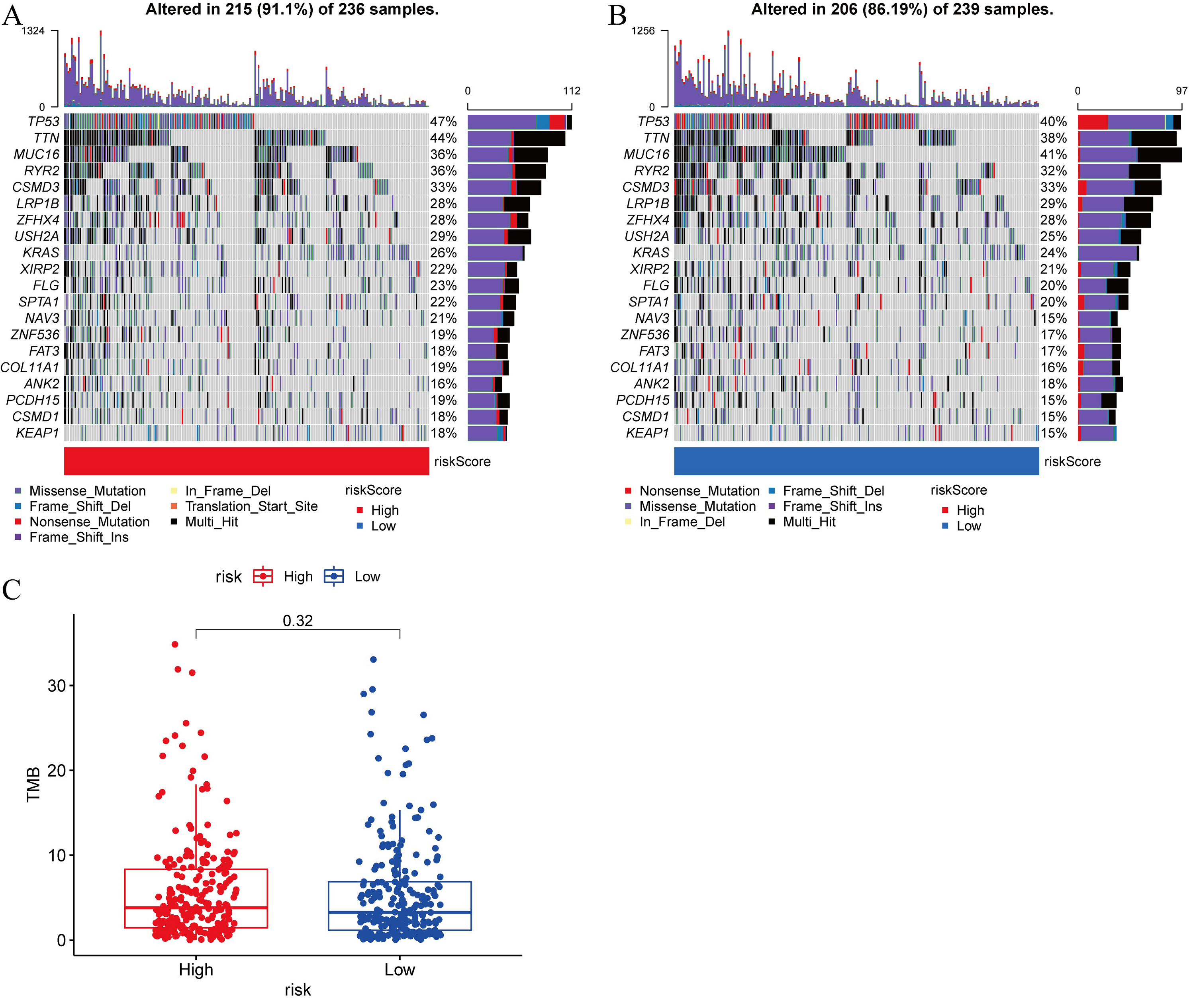

Supplement: Supplemental Information 4 — The waterfall charts of the top 20 mutant genes in low-risk group (A) and high-risk group (B). (C) The TMB between the high- and low-risk groups. [file peerj-09-12275-s004.png]
