# Supplementary material for: Prognostic model of AU-rich genes predicting the prognosis of lung adenocarcinoma
Source: PeerJ. 2021 Oct 8;9:e12275. doi: 10.7717/peerj.12275 (PMC8504460; doi:10.7717/peerj.12275)
Supplement: Supplemental Information 5 [file peerj-09-12275-s005.docx]

**Table S1.** **Chi-square test for the total mutation and the top 3 genes (TP53, TTN, MUC16) with the highest mutation frequency.**

| **Gene** | **Group** | | ***P* value** |
| --- | --- | --- | --- |
|  | **High-risk** | **Low-risk** |  |
| Total mutation | 215 | 206 | 0.092 |
| Total wild | 21 | 33 |  |
| TP53 mutation | 111 | 96 | 0.131 |
| TP53 wild | 125 | 143 |  |
| TTN mutation | 104 | 91 | 0.184 |
| TTN wild | 132 | 148 |  |
| MUC16 mutation | 85 | 98 | 0.263 |
| MUC16 wild | 151 | 141 |  |
